# Association between progranulin serum levels and dietary intake

**DOI:** 10.1371/journal.pone.0202149

**Published:** 2018-08-17

**Authors:** Bruna Bellincanta Nicoletto, Roberta Aguiar Sarmento, Elis Forcellini Pedrollo, Thaiana Cirino Krolikowski, Luis Henrique Canani

**Affiliations:** 1 Post Graduate Medical Sciences Program: Endocrinology, Faculdade de Medicina, Universidade Federal do Rio Grande do Sul (UFRGS), Porto Alegre, Brazil; 2 Nutrition Course, Área do Conhecimento de Ciências da Vida, Universidade de Caxias do Sul (UCS), Caxias do Sul, Brazil; 3 Nutrition Course, Faculdade de Medicina, Universidade Federal do Rio Grande do Sul (UFRGS), Porto Alegre, Brazil; 4 Division of Endocrinology, Hospital de Clínicas de Porto Alegre, Porto Alegre, Brazil; University of Illinois, UNITED STATES

## Abstract

**Introduction:**

Progranulin (PGRN) is secreted by adipose tissue and has been linked to obesity, insulin resistance and type 2 diabetes mellitus. There is evidence that a high fat diet increases PGRN expression in rodent adipose tissue. In humans, the relationship between diet composition and concentration of PGRN is still unknown.

**Objective:**

To investigate the association between dietary intake and serum PGRN levels.

**Methods:**

This is an exploratory cross-sectional study including 85 subjects. Demographic, clinical, laboratory and anthropometric data were collected. Serum PGRN was determined by enzyme-linked immunosorbent assay after overnight fasting. Dietary intake was assessed by food frequency questionnaire validated for Brazilian southern population. Focused principal component analyses (FPCA) was used to verify the association of dietary components and food groups with PGRN levels. Sensitivity analyses were performed including only subjects with reporting according to the Goldberg and Black cut-offs of energy intake-energy expenditure ratio between 0.76 and 1.24.

**Results:**

The median PGRN was 51.96 (42.18 to 68.30) ng/mL. Analyzing all sample, the FPCA showed no association of serum PGRN with total energy, protein, carbohydrate, fat and its types, fiber intake and dietary glycemic index; but a significant and positive association between solid fats and PGRN levels (p<0.05). Including only subjects with reporting according cut-off of energy intake-energy expenditure ratio between 0.76 and 1.24, FCPA showed significant and positive association of serum PGRN with saturated fatty acids and solid fats intake (p<0.05). In this subgroup, PGRN correlated with saturated fatty acids (r = 0.341; p = 0.031). Solid fats intake was independently associated to serum PGRN (beta = 0.294; p = 0.004) in multivariate model.

**Conclusion:**

The dietary intake of solid fats, mainly represented by saturated fatty acids, is associated to serum PGRN concentration in human subjects.

## Introduction

Progranulin (PGRN), also known as proepithelin, granulin-epithelin precursor, acrogranin or PC-cell derived growth factor [1, 2], is a 68–88 kDa protein expressed in many cell types, including epithelial and immune cells, neurons and adipocytes [3]. It plays several functions in the body, acting as a growth factor involved in wound repair, tissue remodeling, cancer growth and survival. Its metabolic functions were recently identified, linking PGRN to obesity, insulin resistance, type 2 diabetes mellitus (T2DM) and inflammation [3–6]. PGRN also has neuronal effects, protecting neurons from premature death [7] and inducing neurite outgrowth [8]. Previous studies have demonstrate that PGRN deficiency in the brain is associated with frontotemporal dementia disease, due to a mutation in the gene encoding PGRN [9].

In hypothalamus, PGRN seems to play a role on food consumption, contributing for appetite suppression and weight loss [10]. Animal studies report that inhibition of PGRN expression in hypothalamus increased food intake and weight gain, while the administration of PGRN significantly suppresses fasting-induced feeding and body weight gain [10]. The action mechanisms of PGRN anorexigenic effects are possibly related to the decreased neuropeptide Y and Agouti-related peptide and increased proopiomelanocortin [10]. In obesity; however, a resistance to PGRN in hypothalamus can lead to hyperphagia [10], similarly to the biological mechanisms previous described for the adipokine leptin [11].

The relationship between food consumption and PGRN levels could be bidirectional. There is evidence that a high fat diet could increase PGRN expression in rodent adipose tissue and liver, as well as inflammatory markers [4]. However, little evidence exists regarding this issue in humans. Therefore, we aimed to investigate the association between dietary intake and serum PGRN levels in subjects attending a general hospital outpatient clinic, in order to test the hypothesis that an unhealthy diet is associated with higher PGRN concentration.

## Materials and methods

### Design and patients

This is an exploratory cross-sectional study, nested in a previous project [12]. The original study included patients with diabetic kidney disease and compared serum and urinary PGRN levels to diabetic and non-diabetic individuals in a sample consisting of 114 subjects attending the Hospital de Clínicas de Porto Alegre (Rio Grande do Sul, Brazil) outpatients clinic. Subjects were recruited between October of 2013 and November of 2014. Exclusion criteria were age below 18 years old and diagnosis of cancer, pancreatitis, acute infections, pregnancy and alcohol or drug abuse. For the present study, individuals with estimated glomerular filtration rate (eGFR) <60 mL/min/1.73m^2^ were also excluded, since PGRN serum levels showed to be dependent on kidney function [12]. Consequently, 91 subjects from previous study [12] were eligible for the present evaluation. Total daily energy consumption above 6000 kcal were also considered for further exclusion [13].

This study was approved by the Ethics Committee of Hospital de Clínicas de Porto Alegre and all subjects received adequate information about the study and gave their written informed consent.

### Clinical, anthropometric and laboratorial assessment

Clinical, anthropometric and laboratorial assessment were previously described [12]. Briefly, a standard questionnaire and review of medical registry were used to access demographic and clinical data. Hypertension was defined by blood pressure ≥140/90 mmHg or antihypertensive medication use, whereas T2DM diagnosis was based on American Diabetes Association criteria [14].

The anthropometry consisted of weight, height, body mass index (BMI) calculation (weight/height^2^) and waist circumference measured at the midpoint between the lowest rib and the iliac crest. All assessments were performed by the same dietitian. Body fat percentage (BF%) and trunk fat (kg) were assessed by a direct segmental multiple-frequency bioelectrical impedance analysis method (InBody 230; Biospace, Seoul, Korea) with the patient fasting, without shoes, wearing light clothing, in a stable condition [15].

Blood samples were taken after 12-hour overnight fasting. The following laboratorial tests were performed: high-sensitivity C reactive protein (hsCRP), fasting plasma glucose, HbA1c, total cholesterol, HDL-cholesterol, triglycerides, proteinuria, albuminuria and serum and urinary creatinine. LDL-cholesterol was calculated using the Friedewald formula when triglyceride levels were lower than 400 mg/dL. The eGFR was assessed by the Chronic Kidney Disease Epidemiology Collaboration (CKD-EPI) equation [16].

Blood was centrifuged, and samples obtained were stored in duplicates at -80°C for later PGRN and IL-6 analysis. The PGRN concentration was determined using the Human Progranulin Quantikine ELISA kit (R&D Systems, Minneapolis, MN, USA), which provide as normal range 33.0–80.8 ng/mL and mean ± SD 51.6 ± 11.5 ng/mL. Assay sensitivity was 0.54 ng/mL and assay range was 1.56–100 ng/mL, whereas the inter-assay coefficient was less than 10%. IL-6 concentration was assessed by Human IL-6 Quantikine ELISA kit (R&D Systems, Minneapolis, MN, USA). The assay sensitivity was 0.7 pg/mL, with a range between 3.12–300 pg/mL and inter-assay coefficient less than 6.5%.

### Dietary intake

Information of food consumption was collected from a quantitative food frequency questionnaire (FFQ) previously constructed and validated in patients from Southern Brazil [17, 18]. FFQ consists of 98 food items grouped into 9 categories: “cereals, tubers, roots, and derivatives”; “vegetables and legumes”; “fruits”; “beans”; “meat and eggs”; “milk and dairy products”; “oils and fats”, “sugars and sweets” and “beverages”, and covered the past 12 months of food intake.

The intake report obtained by the FFQ was converted into daily consumption for the nutritional composition calculation. First, the Brazilian Food Composition Table (TACO, from *Tabela Brasileira de Composição de Alimentos*) [19] was consulted. When some food was not provided at TACO, second and third option were, respectively, Food Composition Table: Support for Nutritional Decision [20] and United States Department of Agriculture (USDA) National Nutrient Database [21]. Dietary components assessed were: total energy, protein, fat and its types, carbohydrate and fiber. Based on the food daily consumption, the dietary glycemic index (GI) of each patient was calculated using international tables [22]. When the GI of foods present in the instruments was not found, data from food with a similar composition were used.

The intake report obtained by the FFQ was also evaluated according to food groups. All foods were estimated in daily consumption (g/day) and were allocated in the following groups: breads (including cereals, tubers, roots, and derivatives), fruits and vegetables, milks (including milk and derivatives), meats, beans, sugars, solid fats (margarine, butter, buttercream, mayonnaise and lard) and vegetable oils (olive, canola, corn and soy oils). Calculations were performed using the syntax of the Statistical Package for Social Sciences version 20.0 program (SPSS, Chicago, IL).

As sensitivity analyses, subjects with acceptable reporting were evaluated. By the way, the term acceptable is proposed to identify individuals who properly reported their energy intake. It is defined by the energy intake-energy expenditure ratio. The Goldberg and Black cut-offs [23–25] were used for this assessment. First, the basal metabolic rate (BMR) was obtained by a direct segmental multiple-frequency bioelectrical impedance (InBody 230; Biospace, Seoul, Korea). The BMR value was multiplied for physical activity index according to the FAO/WHO/UNU recommendation [26], to estimate the energy expenditure. Then, energy intake obtained by FFQ was divided by energy expenditure. Patients with values between 0.76 and 1.24 were considered as acceptable-reporting. Four patients did not have bioelectrical impedance evaluation and were not included in this analysis.

### Statistical analyses

Energy and nutrient intake data were adjusted before analyses for energy intake according to the residual method [27] using the Statistical Package for Social Sciences version 20.0 program (SPSS, Chicago, IL).

Focused principal component analyses (FPCA) were performed to verify the association of dietary components with PGRN levels, using the R version 3.2.3 software (R Development Core Team, Vienna, Austria, available at http://www.r-project.org/). First, we ran FPCA considering dietary factors: energy (kcal/day), protein (g/day), carbohydrates (g/day), fats (g/day) and fiber (g/day) intake and dietary GI. Then, we ran FPCA with lipids types: saturated fatty acids (SFA), monounsaturated fatty acids (MUFA), polyunsaturated fatty acids (PUFA), trans fatty acids (TFA) and cholesterol, all in grams per day. We also ran an FPCA considering food groups (g/day): breads, fruits and vegetables, milks, meats, beans, sugar, solid fats and vegetable oils. As sensitivity analyses, the same was ran using only patients with acceptable reporting. The graphs generated by FPCA presents a red circle delimiting the statistical significance of the correlation at the 5% level. Dots inside the red circle show significant associations with the focus variable (PGRN), the green ones have positive association, while the yellow dots have negative.

The median serum PGRN was used as cutoff to stratified sample into two groups. Clinical, anthropometric and biochemistry variables were analyzed using the Statistical Package for Social Sciences version 20.0 program (SPSS, Chicago, IL). After assessing normality of continuous variables by the Shapiro Wilk test, the groups were compared by Student’s T or Mann-Whitney tests, as appropriate. Data with normal distribution are presented as mean ± standard deviation (SD), whereas data with asymmetric distribution are presented as median (interquartile range, P25-P75). Categorical variables were compared among groups by Chi-square test and they are reported as absolute numbers and percentages.

The correlation between serum PGRN and dietary variables was tested by Pearson’s correlation coefficient. Multivariate linear regression analyses were performed using serum PGRN as dependent variable. First, univariate regressions were run for variable with significant difference between groups divided according median PGRN. Then, variables with p<0.10 at univariate regression were included in the multivariable model. Only valid cases were included in each analysis. The level of statistical significance was established as 5%.

## Results

### Clinical characteristics

Of 91 eligible patients from previous study [12], 6 had no dietary assessment. No one had energy daily consumption over 6000 kcal, resulting in 85 patients included in the present study. Of this, 47 (55.3%) were women and the mean age was 60.9 ± 8.6 years. The median BMI was 30.9 (26.6–35.3) kg/m^2^, with a high prevalence of obesity in total sample (55.3% with obesity, 34.1% overweight and 10.6% normal BMI). Sixty-five subjects (76.5%) had T2DM, and the diabetes duration was 13 (7–21) years.

The median serum PGRN was 51.96 (42.18 to 68.30) ng/mL in all sample. Stratifying by PGRN median in two groups, there was significant difference in T2DM prevalence, BMI, waist circumference, BF%, trunk fat and hsCRP, all being higher in patients group with higher serum PGRN (Table 1).

**Table 1:** Demographic, clinical and anthropometric characteristics of study subjects.

	PGRN ≤ 51.96 ng/mL (n = 43)	PGRN > 51.96 ng/mL (n = 42)	P value
Serum PGRN (ng/mL)	42.6 ± 6.8	69.9 ± 13.1	-
Age (years)	61.5 ± 8.9	60.3 ± 8.4	0.516
Male gender, n (%)	20 (46.5)	18 (42.9)	0.904
Type 2 diabetes mellitus, n (%)	28 (65.1)	37 (88.1)	0.025
Hypertension, n (%)	31 (73.5)	39 (92.9)	0.098
Systolic blood pressure (mmHg)	133 (125–148)	140 (120–149)	0.768
Diastolic blood pressure (mmHg)	80 (69–90)	80 (79–90)	0.177
Obesity, n (%)	17 (39.5)	30 (71.4)	0.003
Body mass index (kg/m^2^)	28.4 (26.2–31.7)	32.5 (28.8–39.3)	0.002
Waist circumference (cm)	101.1 ± 10.6	109.3 ± 14.8	0.004
Body fat %	34.1 ± 9.0	39.0 ± 9.9	0.022
Trunk fat (kg)	14.5 ± 4.6	17.7 ± 5.8	0.006
Fasting plasma glucose (mg/dL)	130.4 ± 57.11	150.4 ± 62.7	0.128
HbA1c (%)	7.59 ± 1.86	7.70 ± 1.56	0.764
Total cholesterol (mg/dL)	183.3 ± 43.6	176.6 ± 35.3	0.435
LDLc (mg/dL)	109.7 ± 37.8	99.0 ± 29.3	0.152
HDLc (mg/dL)	39.0 (35.0–46.0)	41.0 (33.0–47.3)	0.573
Triglycerides (mg/dL)	135.0 (97.0–179.0)	144.0 (102.8–216.5)	0.496
hsCRP (mg/dL)	2.10 (0.93–4.98)	3.65 (2.59–12.33)	0.002
IL-6 (pg/mL)	3.12 (3.12–3.18)	3.12 (3.12–4.39)	0.335
eGFR (mL/min)	97.3 ± 17.1	98.1 ± 17.2	0.827

Data are expressed as mean ± standard deviation, median (interquartile range, P25-P75) or number of patients (%). PGRN: progranulin; hsCRP: high-sensitivity C reactive protein; IL-6: interleukin-6; eGFR: estimated glomerular filtration rate

### Dietary intake and PGRN

The nutrients and dietary intake of all subjects are shown in Table 2. The median total energy intake was 1897 (1394–2409) kcal, distributed in proteins, fats and carbohydrates (Table 2).

**Table 2:** Food intake of study subjects.

	All sample (n = 85)
Total energy (kcal/day)	1897 (1394–2409)
Protein (g/day)	80.8 ± 15.9
Protein (g/kg/day)	0.99 ± 0.24
Fat (g/day)	73.8 ± 16.2
Saturated fat (g/day)	21.8 (19.1–24.2)
Monounsaturated fat (g/day)	22.4 (19.1–25.9)
Poliunsaturated fat (g/day)	19.2 ± 7.2
Trans fat (g/day)	1.6 (1.1–2.2)
Cholesterol (g/day)	232.7 (194.8–286.0)
Carbohydrate (g/day)	254.5 ± 44.2
Fiber (g/day)	24.5 ± 7.7
Diet Glycemic Index (%)	47.4 (44.6–51.2)
**Food groups**	
Breads (g/day)	255 (212–394)
Fruits and vegetables (g/day)	605 (428–915)
Milks (g/day)	229 (118–345)
Meats (g/day)	127 (88–187)
Beans (g/day)	66 (272–126)
Solid fats (g/day)	5 (1–11)
Vegetable oils (g/day)	20 (13–30)
Sugars (g/day)	34 (10–120)

Data are expressed as mean ± standard deviation or median (interquartile range, P25-P75).

**Fig 1:**
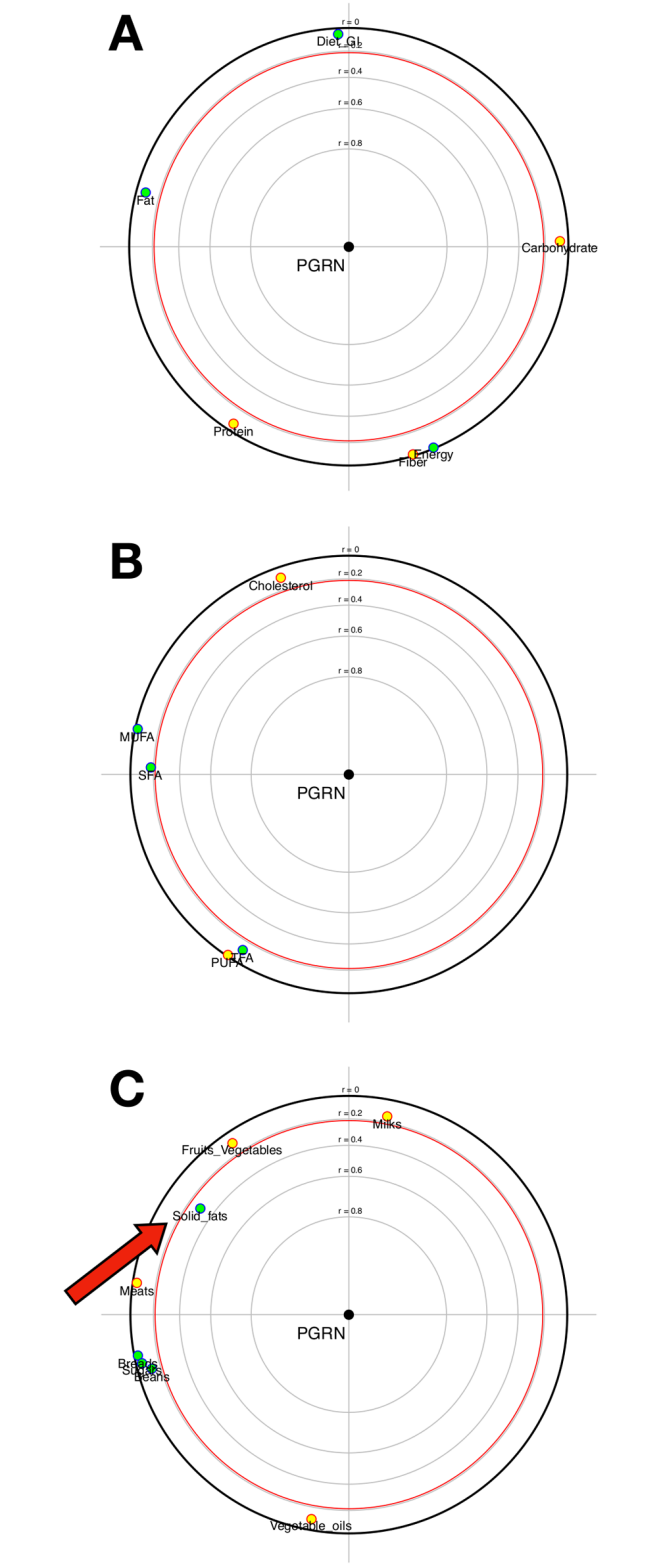
Focused principal component analysis: Progranulin and dietary intake (all sample, n = 85). **A)** Dietary factors: energy (kcal/day), protein (g/day), carbohydrate (g/day), fat (g/day) and fiber (g/day) intake and dietary glycemic index. **B)** Dietary factors (g/day): SFA (saturated fatty acids), MUFA (monounsaturated fatty acids), PUFA (polyunsaturated fatty acids), TFA (trans fatty acids) and cholesterol. **C)** Dietary factors (g/day): breads, fruits and vegetables, milks, meats, beans, solid fats, vegetable oils, and sugar.

The FPCA regarding association of dietary components with PGRN levels is presented in Fig 1. There was no association of PGRN serum levels with total energy, protein, carbohydrate, fat, fiber intake and dietary GI (Fig 1A). Also, no statistical difference was found in the analysis of lipid types (Fig 1B). However, the analysis of food groups showed a significant and positive association between solid fats intake and serum PGRN levels (Fig 1C).

**Fig 2:**
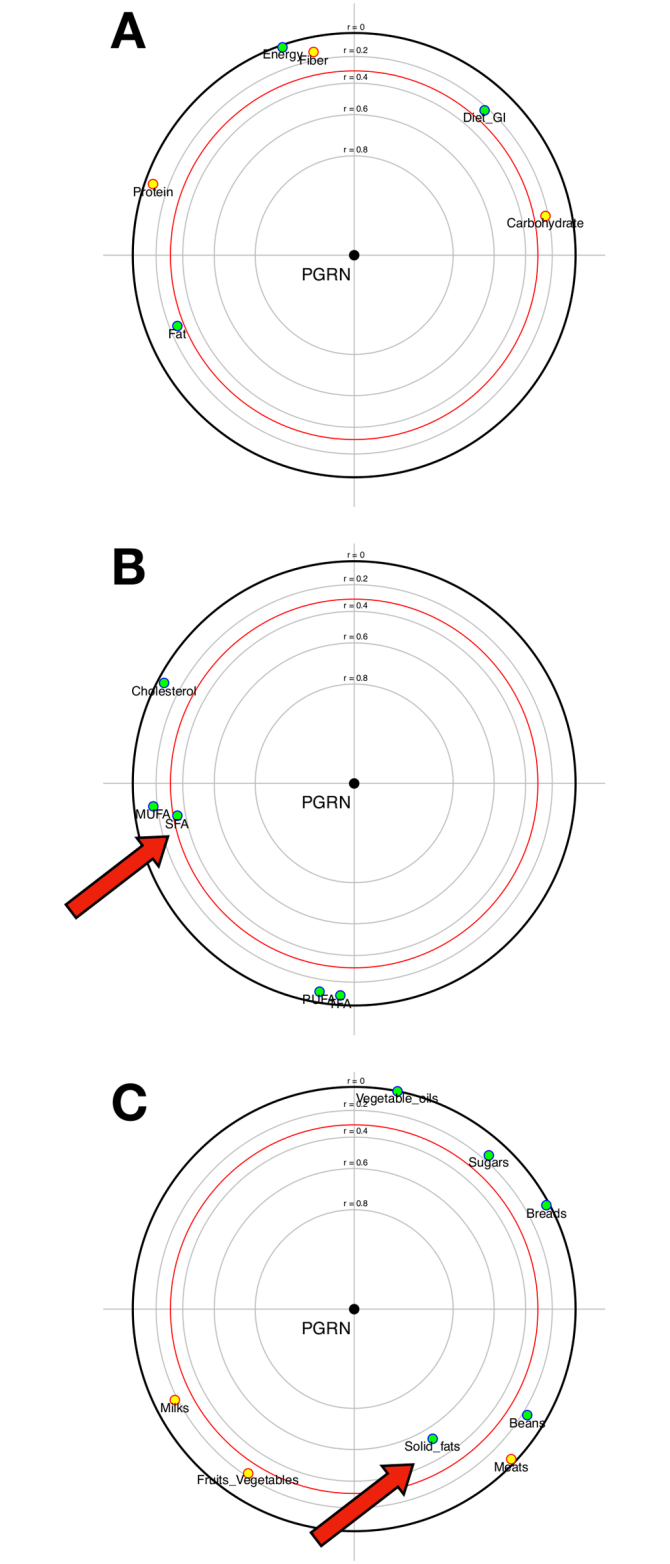
Focused principal component analysis: Progranulin and dietary intake (acceptable reporters, n = 40). **A)** Dietary factors: energy (kcal/day), protein (g/day), carbohydrate (g/day), fat (g/day) and fiber (g/day) intake and dietary glycemic index. **B)** Dietary factors (g/day): SFA (saturated fatty acids), MUFA (monounsaturated fatty acids), PUFA (polyunsaturated fatty acids), TFA (trans fatty acids) and cholesterol. **C)** Dietary factors (g/day): breads, fruits and vegetables, milks, meats, beans, solid fats, vegetable oils, and sugar.

The sensitivity analysis, including only subjects with acceptable reporting is presented in Fig 2. Forty patients were analyzed. There was no association of PGRN serum levels with total energy, protein, carbohydrate, fat, fiber intake and dietary GI (Fig 2A). However, a significant and positive association was found between SFA intake and serum PGRN levels in this subset of patients (Fig 2B). Moreover, the positive association of solid fats with PGRN was maintained in this analysis (Fig 2C). No other association was found statistically significant. The median PGRN concentration in patients with acceptable reporting was 52.28 (42.59–68.71). There was no difference between groups when stratified by median PGRN for clinical variables, except for hypertension prevalence (78.9% vs. 100%; p = 0.042), that was higher in patients with increased serum PGRN.

### Association of SFA intake, solid fats and PGRN

Solid fats intake was positively correlated to serum PGRN levels (r = 0.302; p = 0.005; n = 85) in all sample. In sensitivity analysis, including only subjects with acceptable reporting, there was a stronger correlation (r = 0.534; p<0.001; n = 40). Moreover, a positive correlation was also observed between serum PGRN and SFA intake in this subset of patients (r = 0.341; p = 0.031; n = 40). No other correlations were observed when tested by Pearson’s correlation coefficient.

In the multivariate linear regression analyses using serum PGRN as dependent variable, the solid fats intake remained independently associated to serum PGRN levels in all sample and also in the subset of acceptable reporting patients (Table 3). The SFA intake, however, was not maintained as a predictor of PGRN concentration (Table 3). Besides the independent variables included in multivariable models, age, hypertension, eGFR and IL-6 were tested in univariable model, however they did not reached p<0.10 and were not included in the multivariable model.

**Table 3:** Multivariable linear models using serum PGRN as dependent variable.

Variable	Beta	95% Confidence Interval	P value
**All sample (n = 85)**			
Solid fats intake (g/day)	0.294	0.177 to 0.887	0.004
Female gender	0.115	-2.984 to 10.892	0.260
Body mass index (kg/m^2^)	0.255	0.136 to 1.244	0.015
hsCRP (mg/dL)	0.181	-0.017 to 0.469	0.068
Type 2 diabetes mellitus	0.169	-1.119 to 14.908	0.091
**Acceptable reporting (n = 40)**			
Solid fats intake (g/day)	0.466	0.290 to 1.153	0.002
Female gender	0.156	-4.687 to 15.685	0.280
Body mass index (kg/m^2^)	0.178	-0.293 to 1.208	0.224
hsCRP (mg/dL)	0.097	-0.841 to 1.601	0.531
Type 2 diabetes mellitus	0.182	-3.600 to 19.460	0.171
**Acceptable reporting (n = 40)**			
Saturated fat intake (g/day)	0.255	-0.134 to 1.435	0.101
Female gender	0.103	-7.752 to 15.005	0.521
Body mass index (kg/m^2^)	0.187	-0.358 to 1.317	0.253
hsCRP (mg/dL)	0.190	-0.586 to 2.076	0.263
Type 2 diabetes mellitus	0.252	-2.001 to 23.956	0.095

hsCRP: high-sensitivity C reactive protein

## Discussion

In the present study, it was possible to explore the relationship between dietary intake and serum PGRN. Previous experimental study provides similar findings. Matsubara et al. [4] reported that mice fed with high fat diet had increased PGRN levels in white adipose tissue and liver. Moreover, mice that encode PGRN presented higher weight gain, fat deposition and insulin resistance than PGRN deficient mice. It is also suggested that PGRN acts enhancing IL-6 expression, impairing insulin signaling [4]. This may be a mechanistic step linking a high fat diet with obesity and insulin resistance. In the present study, the fat component was also related to PGRN levels, through solid fats and/or SFA intake.

SFA intake could also been linked to obesity and insulin resistance through lipotoxicity [28]. The elevation of free fatty acids concentration promotes changes in the metabolism. Lipid accumulation in non-adipose tissues leads to deleterious effects, including inflammation and insulin resistance [29], an environment also marked by high concentrations of PGRN [6]. The excess of SFA intake could be involved in this context [29], since it was previously demonstrated its association with impaired glucose metabolism [30].

The main hypothesis for the association between SFA and serum PGRN could possibly be related to the ability of SFA increase inflammation [31]. It was previously reported that SFA intake activate NLRP3 (NOD-like receptors family pyrin domain containing 3) inflammasome, a group of proteins involved in the control of inflammatory cytokines production [31–33]. The PGRN, in its turns, is a pro-inflammatory molecule [6]. By the way, SFA also increases inflammation in the gut microbiome, raising lipopolysaccharide (LPS) levels [34]. In addition, it has been reported that SFA influence the cytokine secretion in adipose tissue [31].

SFA intake has also been related to increased cardiovascular diseases [35, 36]. The main recommendation has been to replace SFA for unsaturated fat acids [35]. Solid fats analyzed in the present study are rich in SFA, including dairy fat (butter) and lard (pork). This group also comprises margarine, a fat rich in TFA, which consumption should be avoided [37]. The association between solid fats and PGRN could be linked to metabolic disorders, once both were previously related to poor outcomes.

Further studies are needed to confirm our findings and elucidate if a reduction in solid fats or SFA intake could contribute to a reduction in PGRN serum concentration. Other evidence [38] reported that a dietary intervention can decrease PGRN levels. Subjects following low-fat, low-carb or Mediterranean diets at the “Dietary Intervention Randomized Controlled Trial (DIRECT)” study were analyzed together up to 24 months. At 6 months, there was a decrease in serum PGRN, and it was sustained at the end of the study, despite some weight regain [38]. The authors report that the PGRN dynamics suggest that this adipokine are directly linked to reduced food intake and/or diet intervention effects, independently of body weight changes [38]. There was no significant differences in PGRN levels between diets, although Mediterranean diet presented numerically lower PGRN values [38]. This diet is well characterized by being rich in fruits and vegetables and replaced SFA for unsaturated fats, such as olive oil, nuts and fish, contributing significantly for health [39]. Other nutritional intervention on reducing PGRN was recently evaluated in a sample of type 2 diabetic kidney disease patients [40]. After eight weeks consuming a probiotic (*Lactobacillus plantarum A7*) soy milk, subjects presented significant reduction in serum PGRN levels when compared to the control group (soy milk only) [40]. The probiotic has beneficial effect on reducing inflammatory adipokine levels [41], so it could be also involved in decreasing PGRN, since it has been reported as an inflammatory molecule [6].

Despite some evidence linking nutritional intervention to reduction in PGRN levels, oral uptake of carbohydrates and lipids does not influence circulating PGRN in a short-term manner [42]. This was verified after 2, 4 and 6 hours of an oral lipid tolerance test and after 1 and 2 hours of oral glucose tolerance test in health individuals. During tests, PGRN concentrations remained unchanged [42].

Although PGRN seems to play an anorexigenic effect, paradoxically it has been suggested in animal models that obesity could be associated with PGRN resistance and it could lead to higher appetite and food intake [10]. Individuals with overweight and obesity mainly comprised the sample population of the present study. Therefore, PGRN signaling at hypothalamus may be compromised. It was previously reported that PGRN concentration are increased in patients with obesity and T2DM [6].

The metabolic effect of PGRN seems to be ambiguous. On the one hand, the pro-inflammatory mechanism links PGRN to obesity, insulin resistance and T2DM. On the other hand, this molecule has been previously related to anti-inflammatory conditions, such as wound repair, psoriasis, arthritis, acute ischemia-reperfusion injury and neurodegenerative diseases [6, 43]. In central nervous system, PGRN has neurotrophic effects and its deficiency is associated to frontotemporal dementia [44]. At the same time, it is known that obesity is associated with increased risk for neurodegenerative disease [45], in this condition; however, PGRN levels are not yet determined.

The PGRN mechanisms of action is still not entirely understood. It has been reported that PGRN binds to tumor necrosis factor receptor-1 (TNFR-1), impairing tumor necrosis factor alfa (TNF-α) binding to its receptor [46, 47]. It could result in anti-inflammatory effect. However, it is also previously reported that activation of TNFR-1 contributes to NLRP3 inflammassome [31]. It is not known how PGRN could be in fact involved in this process. Possibly it plays different functions in distinct tissues and metabolic settings [6]. In the present study, however, it was not possible to establish an association of dietary intake, PGRN levels and distinct metabolic conditions, however, they should be considered when the current results are taken into account.

This study provides a comprehensive evaluation of the relationship between dietary intake and serum PGRN. However, there are some limitations. First, for been an exploratory study, there was no prior evidence to conduct sample size calculation, and the sample is relatively small. Second, the sample population in its majority is composed by subjects with obesity and T2DM, limiting the conclusions to those subjects. Third, there are some limitations on estimating nutrient / food intake. The FFQ is a well stablished method, and it is the most used for dietary pattern evaluation [48]. However, there is limitation regarding memory and estimation of the portion size consumed. In the present study, there were a medium percentage of acceptable reporters, finding corroborating by one study that used the same method [23]. Other studies found even less percentage of adequate reporters [49–51]. Despite it is a cross-sectional study, the FFQ refers to the last 12 months; however, it does not allow an investigation of a causative role between diet intake and increment of PGRN serum concentration. Besides, it is important to emphasize that this study evaluated dietary pattern, which has the advantage of assessing effects of the whole diet. In addition, the FPCA method allow us to visualize dietary patterns based on a given focus variable [52].

In conclusion, intake of solid fats, mainly represented by SFA, seems to be associated to serum PGRN concentration. Several hypothesis on causality could be raised, such as lipotoxicity [28], the impact of SFA on inflammasome activation [31], gut microbiome [34] and cytokine secretion in adipose tissue [31]. Experimental studies regarding the role of SFA on these mechanisms and then the impact on PGRN concentration could contribute for further scientific knowledge. Further studies with a larger sample size and distinct populations are necessary to investigate the association of diet intake and PGRN levels in humans. Moreover, prospective studies with a longitudinal design could contribute for causal inferences. A study with monitoring of dietary intake or a randomized clinical trial with diet handling could better demonstrate the impact of diet on serum PGRN levels.
